# Improvement of Chronic Idiopathic Urticaria With Levothyroxine: A Case Report and Review of Literature

**DOI:** 10.7759/cureus.3209

**Published:** 2018-08-27

**Authors:** Amir Shahbaz, Sri Harshavardhan Senapathi, Kashif Aziz, Muhammad Umair, Amna Khalid, Issac Sachmechi

**Affiliations:** 1 Internal Medicine, Icahn School of Medicine at Mount Sinai/Queens Hospital Center, New York, USA; 2 Medicine, Queens Hospital Center, Jamaica, USA; 3 Internal Medicine, Icahn School of Medicine at Mount Sinai Queens Hospital Center, New York, USA; 4 Internal Medicine, Icahn School of Medicine at Mount Sinai Queen Hospital Center, West Hempstead, USA; 5 Internal Medicine, Icahn School of Medicine, Mount Sinai/Queens Hospital Center, New York, USA

**Keywords:** chronic urticaria, thyroid auto-antibodies, levothyroxine

## Abstract

There is an increased association between chronic urticaria (CU) and thyroid autoantibodies. We presented a case of a patient suffering from CU and newly diagnosed Hashimoto’s thyroiditis in which levothyroxine therapy completely resolved the symptoms of urticaria. A 58-year-old female with a past medical history of recurrent urticaria presented with complaints of fatigue and weight gain. Laboratory investigations showed a high thyroid stimulating hormone (TSH) level and a low free thyroxine (T4) level. She had elevated levels of antithyroid peroxidase (anti-TPO) and anti-thyroglobulin antibodies (anti-TG). She was diagnosed as a case of Hashimoto's thyroiditis and started on levothyroxine therapy. She noticed that her uncontrolled recurrent urticaria started to get better and after a few months of therapy, she stopped taking her topical ointments and antihistamines. The reason for the association between positive serological tests for thyroid autoimmunity and CU is unclear. The resolution of chronic urticaria with levothyroxine in our patient with Hashimoto's thyroiditis suggests a common underlying mechanism between the two pathologies.

## Introduction

Chronic urticaria (CU) is defined as recurrent episodes of urticaria, at least twice a week, occurring for six weeks [1]. Hashimoto’s thyroiditis or autoimmune hypothyroidism is the most common cause of hypothyroidism and characterized by the production of thyroid auto-antibodies against thyroid peroxidase and thyroglobulin [2]. There is an increased association between CU and thyroid auto-antibodies as compared to the general population [3]. We present a case report of a patient suffering from CU and Hashimoto’s thyroiditis whose symptoms of urticaria completely resolved with levothyroxine therapy.

## Case presentation

A 58-year-old female with a past medical history of hypertension, diabetes mellitus type 2, hyperlipidemia, vitamin D deficiency, obesity, allergic rhinitis, and uncontrolled recurrent urticaria presented to the clinic with fatigue and weight gain. Review of her medical records showed that her vitals were in the normal range, with blood pressure ranging from 132/70 mmHg, pulse rate 72/min, and weight 210 lbs with a body mass index (BMI) of 38.1 kg/m2. She denied smoking and alcohol intake. She was taking metformin 500 mg twice daily, rosuvastatin 50 mg, hydrochlorothiazide 25 mg, antihistamines, and nystatin-triamcinolone topical 100,000 units/G-0.1% ointment.

Laboratory investigations showed raised thyroid stimulating hormone (TSH) levels as 14 mlU/ml and low levels of free thyroxine (FT4) as 0.4 ng/dl. hemoglobin A1c (HbA1c) was 6.1, eosinophil count was raised 6.5% (0%-5% normal) and eosinophils (absolute) 0.53x103 (N: 0.0-0.4x103), high antithyroid peroxidase antibodies (anti-TPO) 250 IU/ml (0.0-35 IU/mL), and antithyroglobulin antibodies (anti-TG) 437 IU/ml (N: <40 IU/Ml).

Based on investigations, she was diagnosed as a case of Hashimoto’s thyroiditis. She was started on 50 mcg levothyroxine therapy, which was raised to 125 mcg to achieve euthyroid levels. She noticed that her uncontrolled recurrent urticaria started to get better, and after six months of levothyroxine therapy, her TSH was 1.77 mlU/ml and T4 level was 1.2 ng/dl, and the recurrent urticaria completely resolved. She quit taking her topical ointments and antihistamines that she had been using for urticaria. She is on regular follow-up every six months for the last two years and is symptom-free since then.

## Discussion

Chronic urticaria (CU) has many possible etiologies. Establishing the cause of urticaria and its complete resolution is not always possible [1]. Anti-FceR1 and, less frequently, anti-IgE auto-antibodies that lead to the activation of mast and basophilic cells caused by chronic autoimmune urticaria [4]. Patients with CU have serological evidence of auto-antibodies against one or more thyroid antigens. The reason for the association between positive serological tests for thyroid autoimmunity and CU is unclear [3].

At the molecular level, TSH has many of the attributes of a cytokine, and it can regulate the immune responses mainly by direct T cell, B cell, and dendritic cell activation. The receptors of thyroid releasing hormone (TSH) and human prolactin expressed on the cells of the immune system. The mononuclear cells, monocytes, and splenocytes release a considerable concentration of serum TSH when treated with TSH releasing hormone. The cytokine receptors, particularly interleukin (IL) IL-1, 2, and 6, and tumor necrosis factor alpha are expressed on the hypothalamic-pituitary loop. When activated, they lead to the inhibition of TSH releasing hormone-induced thyroid stimulating hormone release. This effect gets amplified in Hashimoto’s thyroiditis. It leads to the continual release of various ILs and cytokines (especially IL-2) by immune cells that could cause an inflammatory state of target organs such as skin [5]. Thyroid hormonal therapy, mainly by TSH suppression, can reduce the symptoms of CU in a patient with Hashimoto’s thyroiditis [6].

The resolution of urticaria after levothyroxine treatment, irrespective of the initial thyroid function status, has been reported by some authors. Aversano et al. studied CU and Hashimoto’s thyroiditis and found 80% of patients had an improvement of urticaria after 12 months of starting levothyroxine therapy [6]. Kiyici S et al. demonstrated an improvement in the clinical symptoms of patients treated with levothyroxine and desloratadine. However, when compared with controls, there was no significant difference between the two groups, suggesting that the improvement seen in CU with levothyroxine treatment might be a placebo effect or the effect of antihistamine treatment [7]. Kandeel et al. found that most patients continued to have relapses of urticaria with no change in the course of their disease after treatment with thyroid hormone, regardless of the presence of hypothyroidism [8].

In Hashimoto’s thyroiditis, thyroid antibodies are more likely present with overt hypothyroidism. Patients with antibody-negative Hashimoto’s thyroiditis had a milder form of hypothyroidism at the time of diagnosis. This could represent an earlier stage or a less aggressive form of Hashimoto’s thyroiditis [9]. Thyroid autoantibodies are polyclonal in normal subjects while in Hashimoto thyroiditis, they are oligoclonal. Anti-TPO antibodies in Hashimoto’s patients can fix, complement, and destroy thyrocytes and act as competitive inhibitors of enzymatic activity. Anti-TPO antibodies from healthy subjects did not interfere with the blocking activity of anti-TPO antibodies from autoimmune thyroiditis patients. Increased levels of anti-thyroid antibodies support the diagnosis of Hashimoto's thyroiditis and the presence of extrathyroidal autoimmune manifestation [10].

The resolution of chronic urticaria with levothyroxine in our patient with Hashimoto’s thyroiditis suggests a common underlying mechanism between the two pathologies. Definitive studies are needed before a strong recommendation for levothyroxine as a treatment for CU in patients can be made.

## Conclusions

We reported a case of a patient in which levothyroxine therapy completely resolved the symptoms of chronic urticaria. Solid pieces of evidence, based on well-conducted, controlled studies, are desirable to determine if there is a therapeutic role for levothyroxine treatment in ameliorating the manifestations of chronic urticaria.
